# A novel association of CCDC80 with gestational diabetes mellitus in pregnant women: a propensity score analysis from a case-control study

**DOI:** 10.1186/s12884-020-2743-3

**Published:** 2020-01-28

**Authors:** Lei Liu, Jiajin Hu, Ningning Wang, Yang Liu, Xiaotong Wei, Ming Gao, Yanan Ma, Deliang Wen

**Affiliations:** 1 Institute of Health Sciences, China Medical University, No.77 Puhe Road, Shenyang, Liaoning Province 110122 People’s Republic of China; 2 Research Center of China Medical University Birth Cohort, China medical university, Shenyang, Liaoning Province 110122 People’s Republic of China; 3 School of Public Health, Dalian Medical University, Dalian, Liaoning Province 116044 People’s Republic of China; 4 School of Public Health, China Medical University, Shenyang, Liaoning Province 110122 People’s Republic of China

**Keywords:** Gestational diabetes mellitus, Biomarker, CCDC80

## Abstract

**Background:**

Gestational diabetes mellitus (GDM) is a growing global epidemic. Our study aims to confirm the association between circulatory coiled-coil domain-containing 80 (CCDC80) in pregnant women with GDM, to investigate the discriminatory power of CCDC80 on GDM, and to explore the relationships between this molecular level and clinical cardiometabolic parameters.

**Methods:**

A 1:2 matched case-control study with 61 GDM patients and 122 controls was conducted using a propensity score matching protocol. All participants were screened from a multicenter prospective pre-birth cohort: Born in Shenyang Cohort Study (BISCS). During 24 and 28 weeks of gestation, follow-up individuals underwent an oral glucose tolerance test (OGTT) and blood sampling for cardiometabolic characterization.

**Results:**

Following propensity score matching adjustment for clinical variables, including maternal age, gestational age, body mass index, SBP and DBP, plasma CCDC80 levels were significantly decreased in patients with GDM when compared with controls (0.25 ± 0.10 vs. 0.31 ± 0.12 ng/ml, *P* = 0.003). Conditional multi-logistic regression analyses after adjustments for potential confounding factors revealed that CCDC80 was a strong and independent protective factor for GDM (ORs < 1). In addition, the results of the ROC analysis indicated the CCDC80 exhibited the capability to identify pregnant women with GDM (AUC = 0.633). Finally, multivariate regression analyses showed that CCDC80 levels were positively associated with AST, monoamine oxidase, complement C1q, LDL-C, apolipoprotein A1and B, and negatively associated with blood glucose levels at 1 h post- OGTT.

**Conclusions:**

Biomarker CCDC80 could be of great value for the development of prediction, diagnosis and therapeutic strategies against GDM in pregnant women.

## Background

Gestational diabetes mellitus (GDM) refers to impaired glucose tolerance with onset or found for the first time in the second or third trimester of gestation, which is a common metabolic disturbance during pregnancy [1, 2]. In global, GDM impacts 3–25% pregnancies and the continuous soared in the incidence of GDM concurs with the increasing prevalence of maternal obesity [1, 3]. With the development of economy and changes in lifestyle, incidences of GDM in China have been rising yearly from 2.3% in 1999 to 6.8% in 2008 and 9.3% in 2012 [4–6]. Maternal exposure to GDM are regarded to be directly associated with adverse perinatal and late-life complications in offspring [7]. A survey indicated that GDM begot over 50% of pregnant women to suffer from type 2 diabetes among five to 10 years after delivery [8]. Furthermore, it was evaluated that approximately 18.4 million live births worldwide were influenced by GDM in 2017 [9]. Particularly, neonates born to pregnant women with GDM are more vulnerable to obesity, metabolic and cardiovascular diseases during childhood and adulthood [10, 11]. Recently, the most concerning is the reciprocation of the cycle of diabetes between mother and child [12].

Adipocytes not only serve as sites of lipids deposition but can also secrete diverse mediators through autocrine, paracrine or endocrine mechanisms, referred to as adipocyte-secreted proteins or adipokines. These released proteins play pivotal functions in energy homeostasis, insulin sensitivity and systemic inflammation [13]. Coiled-coil domain-containing 80 (CCDC80, also known as DRO1 and URB), a novel adipokine, was originally identified as up-regulated in brown adipose tissue of mildly obese mice [14]. Contrastingly, CCDC80 down-regulation was discovered in white adipose tissue in ob/ob, KKAy and diet-induced obesity mouse models [15]. Previous mechanism studies have provided evidence that CCDC80 plays dual roles in adipogenesis through modulating Wnt/β-catenin signaling, C/EBPα and PPARγ expression [16]. In addition, CCDC80 knockout mice display hyperglycemia, decreased glucose tolerance and impaired insulin secretion in mice fed a high-fat diet [17]. Therefore, CCDC80 might be a novel regulator of energy and glucose metabolism in mice. Meanwhile, a small population-based epidemiological survey has demonstrated that CCDC80 may be a component of the obesity-linked secretome in visceral adipose tissue and whose blood levels are connected to glucose intolerance and low-grade inflammation related chronic complications [18]. And, Li et al. found that serum CCDC80 was negatively correlated with fasting blood glucose (FBG) in overweight and obesity subjects [19]. Nonetheless, whether this molecular is linked to the risk of GDM in pregnant women is less clear.

Hereby, the aims of this study was to estimate the circulatory CCDC80 concentration in pregnant women with GDM in a propensity score matching (PSM) case-control study, to examine the discriminatory power of CCDC80 on GDM, and to elucidate the relationships between the CCDC80 level and clinical cardiometabolic parameters.

## Methods

### Ethics statement

This study was approved by the ethics committee of the China Medical University. Each participant signed an informed consent form. All methods were performed in accordance with the Declaration of Helsinki and relevant guidelines.

### Study design and subjects

A observational, hospital-based, case-control study was carried out to compare the blood CCDC80 concentrations in patients with GDM with scoring propensity matched control subjects from a multicenter prospective pre-birth cohort: Born in Shenyang Cohort Study (BISCS) as described elsewhere [20]. Singleton pregnant women who met the following eligibility criteria were selected from a pool of 1260 Chinese pregnant women: (1) participants obtained fasting blood samples between 24 and 28 weeks during pregnancy; (2) women without pre-existing diabetes mellitus; (3) participants without missing or uncompleted recorded information; (4) no current regular medications.

### Data collection and diagnostic criteria of GDM

The maternal demographic characteristics (self-reported information) was extracted from Maternal and Child Health Handbook. Between the 24th and 28th weeks’ gestation, pregnant women were informed to return to the initial hospital in their fasting state for repeat physical examination. For 2-h 75 g oral glucose tolerance test (OGTT) was performed once in follow-up individuals. Venous blood samples were collected at 0 (fasting), 1 and 2 h after a 75 g glucose load. The blood glucose levels were determined by a biochemical analyzer (ARCHITECT c1600, Japan). We followed the diagnostic criteria of GDM proposed by the Ministry of Health (MOH) of China (fasting blood glucose ≥5.1 mmol/L or 1 h blood glucose of OGTT ≥10.0 mmol/L or 2 h blood glucose of OGTT ≥8.5 mmol/L) [21]. Based on the criteria, subjects were divided into control and GDM groups.

### Blood sample collection and treatment

Elbow venous blood samples after 12-h fasting were drawn and preserved with blood collection tubes containing EDTA (Becton Dickinson and Co., UK). The blood samples were immediately centrifuged at 3000 rpm for 10 min at 4 °C and the EDTA-plasma was aliquoted and stored at − 80 °C until assayed all at once.

### Clinical and biochemical measurements

The plasma content of aspartate aminotransferase (AST), alanine aminotransferase (ALT), monoamine oxidase (MAO), creatinine, complement C1q, triglyceride, cholesterol, high-density lipoprotein cholesterol (HDL-C), low-density lipoprotein cholesterol (LDL-C), Apolipoprotein A1 (apoA1) and B (apoB) were detected by an automatic biochemical analyzer (ARCHITECT c1600, Japan), and C-reactive protein (CRP) was analyzed by an automatic special protein analyzer (Beckman Coulter image 800, USA). The levels of secreted IL-6 were measured using an automatic electrochemiluminescence immunoassay system (Roche cobas 6000, Switzerland).

Plasma CCDC80 was detected by monoclonal antibody based commercial enzyme-linked immunosorbent assay (ELISA) kit (cloud-clone corp., USA) with intra- and interassay sample replicant coefficient of variability (CV) of < 10% and < 12%, respectively. Measurements were conducted in duplicates by a single observer to minimize the observer variation.

### Statistical analysis

Propensity score matching (PSM) was utilized in this case-control study to minimize selection bias and to decrease potential confounders with a 1: 2 matching protocol. Matching factors included maternal age, gestational age, body mass index (BMI), systolic blood pressure (SBP) and diastolic blood pressure (DBP). The model was appraised by the Hosmer-Lemeshow goodness-of-fit test for logistic regression analysis [22]. We adopted the nearest neighbour score matching principle.

Continuous and categorical variables were reported as the mean ± standard deviation (SD) and frequencies (percentages), separately. And unadjusted comparisons among the patient group and the control group were performed for significance using Student’s t-test and chi-squared (χ^2^) tests. CCDC80 z-score was calculated adopting the formula z = (x-μ)/σ, where x is the CCDC80 blood value, while μ and σ are the mean and SD, separately. After PSM, a conditional multi-logistic regression analysis after adjustments for potential confounding factors was implemented to determine the connection between plasma CCDC80 levels and the presence of GDM. Odds ratios (ORs) and 95% confidence intervals (CIs) were presented. Afterwards, the area under the receiver operating characteristic (ROC) curve (AUC) were measured to observe the discriminatory performance of CCDC80 for the GDM risk. The interrelations between circulating CCDC80 levels and other clinical or laboratory parameters were discovered by univariate and multivariate regression analyses. Statistical significance was defined as *P* < 0.05. All statistical analyses were performed using Stata SE 12.0 and SPSS 20.0.

## Results

### Findings in the case-control study

A final total of 61 GDM patients and 122 paired controls were enrolled into the study. Demographics characteristics of the study subjects are shown in Table 1. Before PSM, maternal age, weight, BMI, FBG, OGTT 1 h, OGTT 2 h and blood pressure was significantly higher in the GDM group than that in the control group. With the use of PSM, there were no significant differences in maternal age, weight, BMI status and blood pressure status between two groups. Therefore, the differences in demographics characteristics between two groups were eliminated following PSM. Table 2 shows the features of the clinical cardiometabolic profiles for both two categories of participants and no significant differences were detected between the two groups in the circulating levels of the clinical cardiometabolic parameters between the two groups.

**Table 1:** Demographic characteristics of study participants

Variables	All cases			Propensity score-matched sets		
	Control (*n* = 252)	GDM (*n* = 61)	*P* value	Control (*n* = 122)	GDM (*n* = 61)	*P* value
Age (years)	29.15 ± 3.96	31.64 ± 4.52	< 0.001^*^	31.42 ± 3.73	31.64 ± 4.52	0.725
Gestational age (weeks)	24.32 ± 1.28	24.43 ± 1.46	0.544	24.63 ± 1.24	24.43 ± 1.46	0.337
Ethnicity						
Han, n (%)	210 (83.3)	51 (83.6)	0.959	107 (87.7)	51 (83.6)	0.447
Others, n (%)	42 (16.7)	10 (16.4)		15 (12.3)	10 (16.4)	
Gravidity						
1, n (%)	131 (52.0)	25 (41.0)	0.201	56 (45.9)	25 (41.0)	0.563
2, n (%)	67 (26.6)	17 (27.9)		37 (30.3)	17 (27.9)	
≥ 3, n (%)	54 (21.4)	19 (31.1)		29 (23.8)	19 (31.1)	
Height (cm)	162.63 ± 4.99	162.74 ± 4.60	0.879	162.85 ± 5.06	162.74 ± 4.60	0.882
Weight (kg)	58.37 ± 10.53	63.00 ± 12.59	0.003^*^	61.67 ± 10.99	63.00 ± 12.59	0.464
BMI (kg/m^2^)	22.05 ± 3.76	23.75 ± 4.48	0.002^*^	23.25 ± 3.91	23.75 ± 4.48	0.434
FBG (mmol/L)	4.44 ± 0.32	5.06 ± 0.54	< 0.001^*^	4.49 ± 0.33	5.06 ± 0.54	< 0.001^*^
OGTT 1 h (mmol/L)	7.09 ± 1.39	9.28 ± 1.52	< 0.001^*^	7.28 ± 1.36	9.28 ± 1.52	< 0.001^*^
OGTT 2 h (mmol/L)	6.52 ± 0.95	8.45 ± 1.37	< 0.001^*^	6.59 ± 0.93	8.45 ± 1.37	< 0.001^*^
SBP (mmHg)	105.38 ± 12.4	111.16 ± 13.9	0.002^*^	108.67 ± 11.9	111.16 ± 13.9	0.211
DBP (mmHg)	67.80 ± 8.3	72.31 ± 9.9	< 0.001^*^	70.44 ± 8.3	72.31 ± 9.9	0.182

*BMI* body mass index, *FBG* fasting blood glucose, *OGTT* oral glucose tolerance test, *SBP* systolic pressure, *DBP* diastolic pressure

*P < 0.05

**Table 2:** Clinical cardiometabolic parameters of study participants

Variables	All cases			Propensity score-matched sets		
	Control (n = 252)	GDM (n = 61)	*P* value	Control (n = 122)	GDM (n = 61)	*P* value
ALT (U/L)	10.33 ± 7.93	9.98 ± 5.35	0.744	9.33 ± 6.63	9.98 ± 5.35	0.503
AST (U/L)	12.60 ± 6.62	11.28 ± 5.21	0.146	12.04 ± 5.61	11.28 ± 5.21	0.376
MAO (U/L)	7.43 ± 2.47	6.98 ± 2.18	0.188	7.60 ± 2.58	6.98 ± 2.18	0.110
Creatinine (umol/L)	37.41 ± 19.62	36.71 ± 7.30	0.784	38.88 ± 27.26	36.71 ± 7.30	0.543
Hemoglobin (g/L)	126.17 ± 13.4	129.18 ± 13.0	0.113	126.34 ± 14.5	129.18 ± 13.0	0.199
Inflammatory marker						
WBC count (× 10^9^/L)	8.74 ± 6.88	8.00 ± 1.94	0.412	8.75 ± 7.35	8.00 ± 1.94	0.435
IL-6 (pg/mL)	2.23 ± 0.90	2.45 ± 0.83	0.074	2.21 ± 0.93	2.45 ± 0.83	0.084
CRP (mg/L)	4.67 ± 3.21	5.22 ± 2.69	0.226	4.90 ± 3.00	5.22 ± 2.69	0.488
Complement C1q (mg/L)	158.5 ± 37.5	157.4 ± 34.4	0.824	163.1 ± 37.5	157.4 ± 34.4	0.322
Lipids						
Triglyceride (mmol/L)	1.85 ± 0.85	2.24 ± 1.41	0.006^*^	2.01 ± 0.97	2.24 ± 1.41	0.197
Cholesterol (mmol/L)	4.39 ± 1.09	4.78 ± 3.92	0.170	4.42 ± 1.08	4.78 ± 3.92	0.346
HDL-C (mmol/L)	1.44 ± 0.42	1.35 ± 0.44	0.146	1.43 ± 0.40	1.35 ± 0.44	0.214
LDL-C (mmol/L)	2.38 ± 0.77	2.22 ± 0.80	0.153	2.37 ± 0.76	2.22 ± 0.80	0.201
Apolipoprotein A1 (g/L)	1.60 ± 0.42	1.58 ± 0.42	0.790	1.62 ± 0.42	1.58 ± 0.42	0.528
Apolipoprotein B (g/L)	0.81 ± 0.24	0.80 ± 0.26	0.782	0.83 ± 0.24	0.80 ± 0.26	0.499

*WBC* white blood cell, *IL-6* interleukin-6, *CRP* C-reaction protein, *ALT* alanine aminotransferase, *AST* Aspartic aminotransferase, *MAO* monoamine oxidase, *HDL-C* high-density lipoprotein cholesterol, *LDL-C* low-density lipoprotein cholesterol

*P < 0.05

**Fig. 1:**
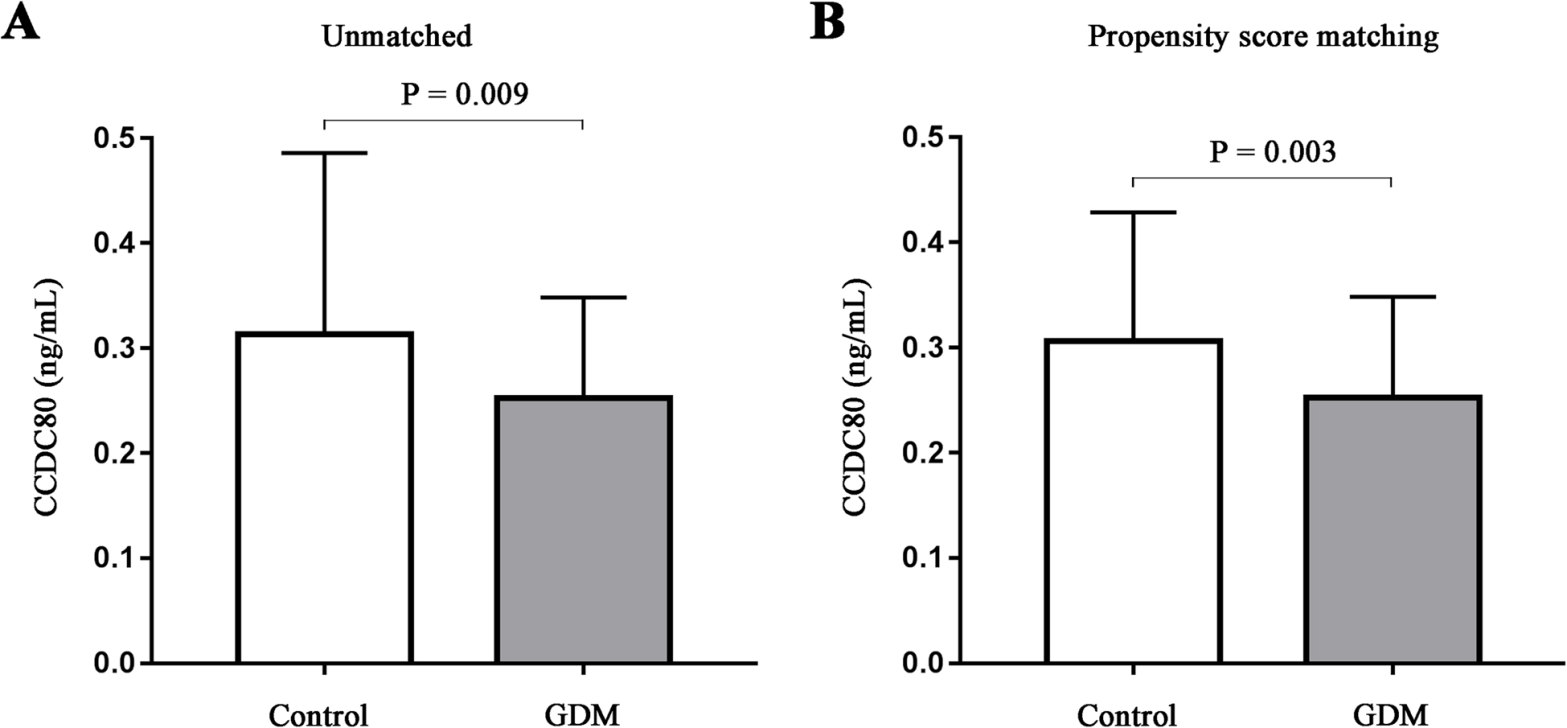
Comparison of circulatory abundance of CCDC80. Plasma CCDC80 levels comparison between GDM patients and controls before PSM (**a**) and after PSM (**b**)

As shown in Fig. 1, regardless before and after PSM, the circulating levels of CCD80 in the GDM subjects was over 19% lower than that in the control group (0.31 ± 0.17 vs. 0.25 ± 0.10, *P* = 0.009; 0.31 ± 0.12 vs. 0.25 ± 0.10, *P* = 0.003, respectively).

### Conditional multi-logistic regression analysis to identify the relationship between the concentration of CCDC80 and GDM

As shown in Table 3, the conditional multi-logistic regression analysis revealed that patients with elevated plasma levels of CCDC80 had a significantly reduced risk of GDM, the unadjusted OR was 0.60 (95% *CI*: 0.42–0.85, *P* = 0.005). We categorized CCDC80 levels into quartile and found that study participants with high CCDC80 (Q4: > 0.361 vs. Q1: ≤ 0.207) had significantly decreased risk of GDM (OR = 0.36, 95% *CI*: 0.14–0.92, *P* = 0.033),and that there was a significant linear trend across categories (trend test, *P* = 0.013). After adjustment for ALT, AST and creatinine (model 2), the adjusted OR was 0.60 (95%*CI*: 0.41–0.89, *P* = 0.011), the linear trend across categories was also significant (trend test, *P* = 0.023). After adjustment for IL-6 and CRP (model 3), the adjusted OR was 0.61 (95%*CI*: 0.42–0.87, *P* = 0.006). Compared with the lowest level group, the OR of highest level CCDC80 was 0.36 (95%*CI*: 0.14–0.94, *P* = 0.036; trend test, *P* = 0.015). After adjustment for triglyceride, cholesterol, HDL-C, LDL-C, apoA1 and apoB (model 4), the adjusted OR for categorized CCDC80 levels was 0.52 (95% *CI*: 0.34–0.78, *P* = 0.002; trend test, *P* = 0.013) (highest vs. lowest quartile). When all participants (before PSM) were enrolled into this analysis, the results were not substantially altered (Additional file 1: Tables S1). Using the conditional multi-logistic regression analysis, we disclosed that CCDC80 was a strong independent predictor of GDM.

**Table 3:**
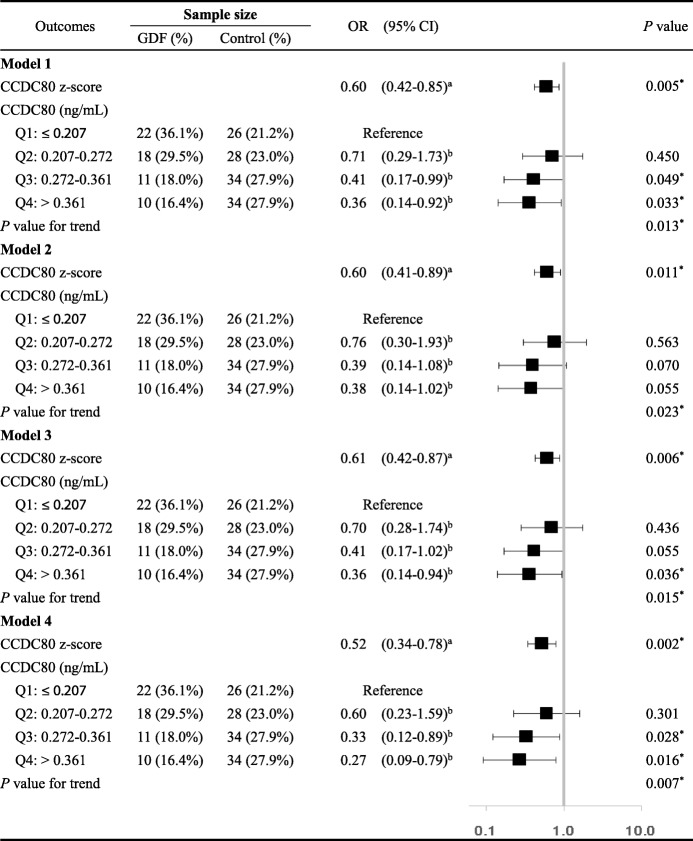
Conditional multi-logistic regression analysis

Model 1: Unadjusted; Model 2: Adjusted for ALT, AST and creatinine; Model 3: Adjusted for IL-6 and CRP; Model 4

Adjusted for triglyceride, cholesterol, HDL-C, LDL-C, apoA1 and apoB. *P < 0.05

^a^ The OR (95% CI) of CCDC80 z-scores using a conditional logistic regression

^b^ The OR (95% CI) referenced to the lowest level using a conditional logistic regression

### ROC curves analysis for the relationship between circulating CCDC80 level and GDM

**Fig. 2:**
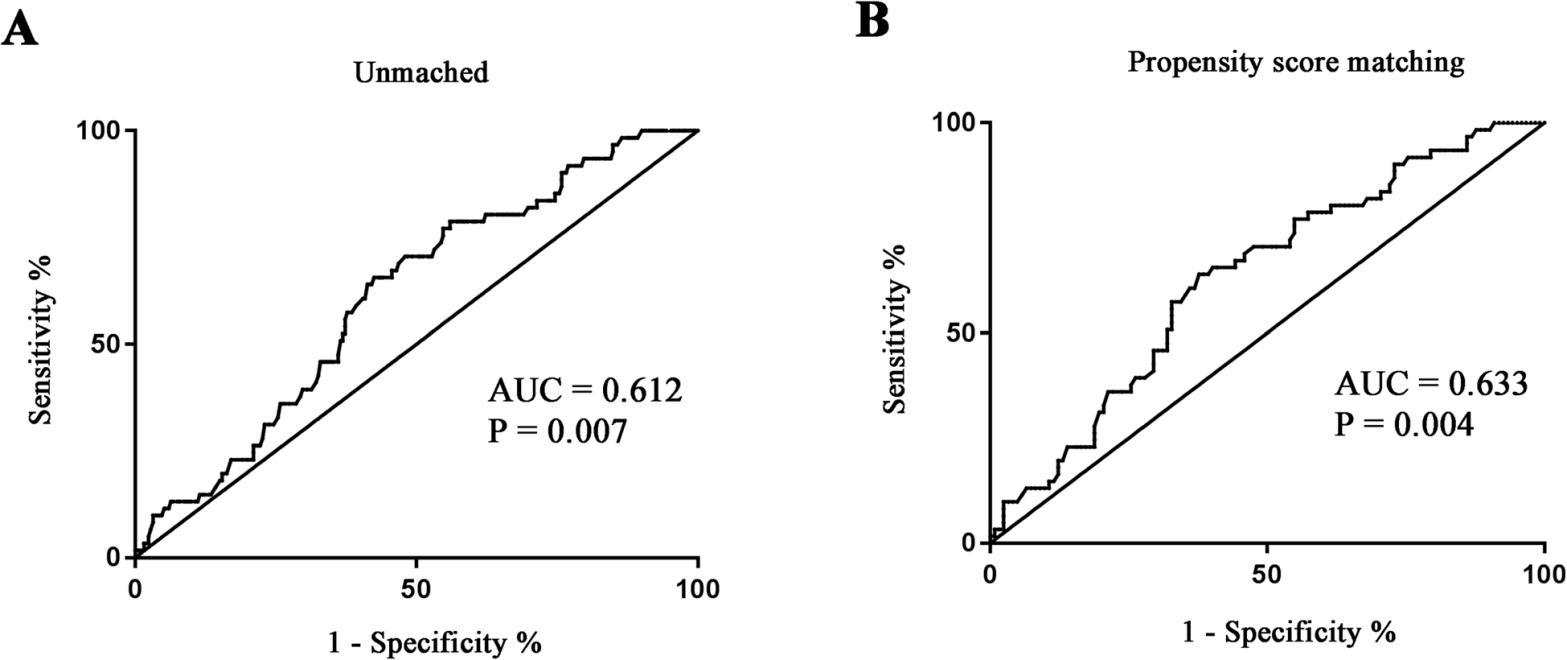
Receiver operating characteristic curve analysis of intercept and slope of circulating CCDC80 levels for distinguish of GDM ROC curve analysis was carried out for unmatched whole samples (A) and PSM subjects.

To esteem the predictive value of CCDC80 for GDM, we undertaken ROC curve analysis. The area under the ROC curve was 0.612 (95%CI: 0.539–0.685) in unmatched full samples (Fig. 2a) and 0.633 (95% CI: 0.550–0.716) in PSM samples (Fig. 2b). We also determined the predictive ability of simple measures routinely available at booking visit (maternal age, gestational age, BMI, SBP and DBP) in the unmatched whole samples. This demonstrated an AUC of 0.724, which increased significantly to 0.744 and 0.735 with the addition of CCDC80 and HDL-C, respectively (Additional file 2). Thus, CCDC80 exhibited acceptable capacity to distinguish the GDM patients from general population.

### The univariate and multivariate associations between CCDC80 and cardiometabolic parameters

As presented in Table 4, among all demographics and clinical cardiometabolic determinants, bivariate correlation analyses showed that CCDC80 levels were positively associated with AST (r = 0.281, *P* < 0.001), MAO (r = 0.252, *P* = 0.001), complement C1q (r = 0.170, *P* = 0.022), LDL-C (r = 0.169, *P* = 0.022), ApoA1 (r = 0.219, *P* = 0.003), ApoB (r = 0.207, *P* = 0.005), and negatively associated with OGTT at 1 h blood glucose (r = − 0.150, *P* = 0.042).

**Table 4:** Univariate and multivariate regression analyses

Variables	Univariate		Multivariate	
	r	*P* value	*β* (95%*CI*)^a^	*P* value
FBG (mmol/L)	− 0.126	0.090	− 0.508 (− 1.129, 0.112)	0.107
OGTT 1 h (mmol/L)	− 0.150	0.042^*^	−2.161 (− 4.269, − 0.052)	0.045^*^
OGTT 2 h (mmol/L)	− 0.138	0.063	− 1.700 (− 3.456, 0.056)	0.058
BMI (kg/m^2^)	0.044	0.556	1.715 (−3.424, 6.854)	0.511
ALT (U/L)	0.093	0.210	5.815 (−1.945, 13.575)	0.141
AST (U/L)	0.281	< 0.001^*^	13.529 (6.886, 20.171)	< 0.001^*^
MAO (U/L)	0.252	0.001^*^	5.312 (2.291, 8.333)	0.001^*^
Creatinine (umol/L)	0.079	0.286	15.493 (−13.229, 44.216)	0.289
Inflammatory marker				
WBC count (× 10^9^/L)	−0.075	0.313	−4.441 (−12.161, 3.278)	0.258
IL-6 (pg/mL)	−0.059	0.425	−0.429 (−1.576, 0.718)	0.462
CRP (mg/L)	−0.079	0.287	−2.095 (−5.757, 1.567)	0.260
Complement C1q (mg/L)	0.170	0.022^*^	52.258 (6.490, 98.025)	0.025^*^
Lipids				
Triglyceride (mmol/L)	0.093	0.210	0.942 (−0.486, 2.370)	0.195
Cholesterol (mmol/L)	0.052	0.485	1.179 (−1.892, 4.250)	0.450
HDL-C (mmol/L)	0.069	0.352	0.255 (−0.272,0.782)	0.341
LDL-C (mmol/L)	0.169	0.022^*^	1.062 (0.103, 2.021)	0.030^*^
Apolipoprotein A1 (g/L)	0.219	0.003^*^	0.784 (0.265, 1.303)	0.003^*^
Apolipoprotein B (g/L)	0.207	0.005^*^	0.432 (0.121, 0.743)	0.007^*^

*BMI* body mass index, *FBG* fasting blood glucose, *OGTT* oral glucose tolerance test, SBP systolic pressure, *DBP* diastolic pressure, *WBC* white blood cell, *IL-6* interleukin-6, *CRP* C-reaction protein, *ALT* alanine aminotransferase, *AST* Aspartic aminotransferase, *MAO* monoamine oxidase, *HDL-C* high-density lipoprotein cholesterol, *LDL-C* low-density lipoprotein cholesterol

^a^ adjusted maternal age and Gestational age. *P < 0.05

To ensure whether plasma levels of CCDC80 were independently correlated with these markers, the multivariate linear regression analysis with adjustment for maternal age and gestational age were performed. The analyses demonstrated that the CCDC80 levels could independently predicted the values of OGTT at 1 h blood glucose (*β =* − 2.161*,* 95%*CI*: − 4.269- -0.052), AST (*β =* 13.529*,* 95% *CI*: 6.886–20.171), MAO (*β =* 5.312, 95%*CI*: 2.291–8.333), complement C1q (*β =* 52.258, 95% *CI*: 6.490–98.025), LDL-C (*β =* 1.062, 95% *CI*: 0.103–2.021), ApoA1 (*β =* 0.784, 95% *CI*: 0.265–1.303) and ApoB (β = 0.432, 95% CI: 0.121–0.743) (Table 4). Additionally, unmatched whole samples were enrolled into this analysis, the results are shown in Additional file 1: Table S2.

## Discussion

In the present study, we determined for the first time that the CCDC80 levels decreased in pregnant women with GDM, compared with normal blood glucose subjects; and this molecule was a strong independent predictor of GDM. So far, the role of CCDC80 in metabolic derangements, especially among pregnant women has been rarely explored. This is one of the first studies to explore the discriminatory power of CCDC80 on the GDM and the relationships between the CCDC80 level and clinical cardiometabolic parameters in pregnant women in a PSM, case-control design study.

CCDC80 has been considered as a multipurpose molecule in vertebrates, mediating diverse developmental processes [23]. Using transcriptional profiling, previous publications have identified that CCDC80 may play a role in tumor inhibition, such as ovarian cancer [24], malignant melanoma [25], thyroid [26] and colorectal carcinoma [27]. CCDC80 is expressed in several types of cells, in particular in preadipocytes and adipocytes, while it is temporarily down-regulated during cell differentiation [16]. Consequently, the role of CCDC80 in metabolism has been gradually recognized in recent years. Tremblay et al. demonstrated that CCDC80 lacking mouse manifested decreased glucose tolerance and insulin sensitivity [17]. Herein, we clarified that the concentration of CCDC80 was lower in pregnancy women with GDM than that of control group suggesting that CCDC80 might have a protective effect on GDM. These results are concordant with previous findings of negative correlation between serum CCDC80 and FBG in adults [19]. Moreover, recent data has indicated that serum level of CCDC80 is linked with glucose clearance and insulin secretion [18]. From the above, we reasoned that circulating CCDC80 may be an effecient biomarker for GDM.

In the present study, conditional multi-logistic regression analyses unveiled that CCDC80 was an independent protective and predictive biomarker for GDM; further, the results of the ROC analysis indicated that CCDC80 displayed the potentiality to identify individuals with GDM (all AUC > 0.5). Taken together, our results collectively suggested that plasma CCDC80 is a candidate clinical indicator for prediction and diagnosis of GDM in pregnant women. Even though the accurate pathophysiological process of CCDC80 on GDM is not yet well-known, findings from mouse models propose that CCDC80 might function to govern glucose equilibrium through Wnt/β-catenin signaling pathway [16, 17]. The role of Wnt/β-catenin pathway signaling in metabolic diseases was well-established. Latest research places WNT signaling pathway in a crucial position in modulating pancreas function, insulin synthesis and secretion [28]. Conjointly, this seems to point out that elevated CCDC80 level is predictive for glucose and insulin sensitivity disturbances, while the specific mechanism pathway needs to be further explored.

Furthermore, univariate and multivariate regression analyses proved that plasma CCDC80 content was negative related with glucose levels at 1 h post- OGTT, but this connection was not remained throughout the OGTT procedure. These findings are in line with prior views of negative correlation between CCDC80 and glucose levels just at 30 min post- OGTT [18]. Besides, our analysis found that CCDC80 was positively correlated with AST and MAO, indicating a positive correlation with liver function. Except for the correlation between serum CCDC80 level and degree of hepatic steatosis observed by Osorio-Conles et al., there is no previous evidence of CCDC80 functioning in liver. Furthermore, component C1q, a marker of innate immune system, was also positively linked to circulating CCDC80 level. Previous studies have illustrated an advantageous effect of component C1q, which triggers activation of the classical pathway, in diabetes mellitus [29, 30]. Finally, we detected that plasma CCDC80 level was positively associated with the dyslipidemia and atherosclerosis marker LDL-C, apoA1 and apoB. These findings are consistent with earlier surveys, which has indicated that blood CCDC80 is positively correlated with atherosclerosis and fasting triglyceride levels [18]. We speculated that circulating CCDC80 might be involved with obesity-related processes. Previous studies have found that CCDC80 promotes the phosphorylation of extracellular regulated protein kinase 1/2 (ERK1/2) [31] that governs lipoprotein lipase, the rate-limiting enzyme for lipid metabolism, expression and activity [32–34]. A number of studies have disclosed that CCDC80 is driven by estrogen and may process a particular feature in the control of body weight and energy homeostasis [35]. Of note, although a higher expression of CCDC80 in visceral adipose tissue of obese subjects, the circulating CCDC80 was not connected to maternal BMI in the current study, either before or after PSM (r = − 0.036, *P* = 0.528 and r = 0.044, *P* = 0.556, respectively). Previous study has yielded similar results [18].

There are also several limitations in our study. First, despite a PSM design has been introduced to minimize the bias, it is impossible all of the relevant confounders were covered in our PSM method. Hence, unidentified potential covariates may still affect the true relations. Second, owing to the cross-sectional data, definite causal relationship between circulating CCDC80 protein and GDM cannot be judged. Further longitudinal studies are required to infer whether this relationship is causal. Third, our blood specimens were collected within the second trimester of gestation, thus we do not have this molecular levels during the first and third trimesters.

Maternal exposure to GDM rises the probability of the development of serious adverse perinatal outcomes and metabolic complications at a later stage of lifespan of the offspring [36], and better understanding the physiopathology and molecular basis of this condition could lead to novel therapeutic strategies. The present study suggests CCDC80 could be vital for the development of therapeutic approaches to battle GDM in pregnant women. It may provide a new target and biomarker for further investigations aimed to explore the longitudinal trend and mechanisms of this molecule during pregnancy.

## Conclusions

In summary, this study demonstrates for the first time that the CCDC80 levels depress in pregnant women with GDM, compared with normal blood glucose subjects; and the CCDC80 is a strong independent predictor of GDM. CCDC80 may be a novel predictor, diagnostic and therapeutic target.

## Supplementary information

**Additional file 1.** Multi-logistic regression analysis for unmatched whole samples.

**Additional file 2.** ROC curves and summaries for all participants using a basic model (including age, gestational age, BMI, SBP, and DBP) and with addition of independent predictors (CCDC80 and HDL-C).

## Data Availability

All data generated or analyzed during this study are included in this published article and its supplementary information files.

## Abbreviations

ALT: Alanine aminotransferase
apoA1/B: Apolipoprotein A1 and B
AST: Aspartate aminotransferase
Bmi: Body mass index
CCDC80: Coiled-coil domain-containing 80
CRP: C-reactive protein
DBP: Diastolic blood pressure
FBG: Fasting blood glucose
GDM: Gestational diabetes mellitus
HDL-C: High-density lipoprotein cholesterol
LDL-C: Low-density lipoprotein cholesterol
MAO: Monoamine oxidase
OGTT: Oral glucose tolerance test
PSM: Propensity score matching
ROC: Receiver operating characteristic
SBP: Systolic blood pressure
