# Supplementary material for: A novel association of CCDC80 with gestational diabetes mellitus in pregnant women: a propensity score analysis from a case-control study
Source: BMC Pregnancy Childbirth. 2020 Jan 28;20:53. doi: 10.1186/s12884-020-2743-3 (PMC6986032; doi:10.1186/s12884-020-2743-3)
Supplement: Supplementary file 1 — Additional file 1. Multi-logistic regression analysis for unmatched whole samples. [file 12884_2020_2743_MOESM1_ESM.docx]

* Additional file 1

**A novel association of CCDC80 with gestational diabetes mellitus** **in pregnant women: a propensity score analysis from a case-control study**

**Authors**

Lei Liu^1^, Jiajin Hu^1,2^, Ningning Wang^2^, Yang Liu^1^, Xiaotong Wei^3^,

Ming Gao^3^, Yanan Ma^3^,Deliang Wen^1,2#^

**Affiliations**

1. Institute of Health Sciences, China Medical University, Shenyang, Liaoning Province 110122, P.R. China
2. Research Center of China Medical University Birth Cohort, Shenyang, Liaoning Province 110122, P.R. China
3. School of Public Health, Dalian Medical University, Dalian, Liaoning Province 116044, P.R. China;
4. School of Public Health, China Medical University, Shenyang, Liaoning Province 110122, P.R. China

**#Corresponding authors**

Correspondence: Professor Deliang Wen, China Medical University, No.77 Puhe Road, Shenyang North New Area, Shenyang, Liaoning Province, 110122, P.R. China.

E-mail: [dlwen@cmu.edu.cn](mailto:dlwen@cmu.edu.cn)

Table S1 Multi-logistic regression analysis for unmatched whole samples

| Outcomes | Sample size | |  | OR (95% CI) | *P* value | |
| --- | --- | --- | --- | --- | --- | --- |
|  | GDF (%) | Control (%) |  |  |  |  |
| **Model 1** | | | | |  | |
| CCDC80 Z-score |  |  |  | 0.549 (0.360-0.838)^a^ | 0.005^*^ |  |
| CCDC80 (ng/mL) |  |  |  |  |  |  |
| ≤ 0.204 | 19 (31.1%) | 60 (23.8%) |  | Reference |  |  |
| 0.204-0.278 | 22 (36.1%) | 57 (22.6%) |  | 1.219 (0.598-2.486)^b^ | 0.586 |  |
| 0.278-0.361 | 10 (16.4%) | 68 (27.0%) |  | 0.464 (0.200-1.077)^b^ | 0.074 |  |
| > 0.361 | 10 (16.4%) | 67 (26.6%) |  | 0.471 (0.203-1.093)^b^ | 0.080 |  |
| *p* value for trend |  |  |  |  | 0.017^*^ |  |
| **Model 2** | | | | |  |  |
| CCDC80 Z-score |  |  |  | 0.493 (0.309-0.785)^a^ | 0.003^*^ |  |
| CCDC80 (ng/mL) |  |  |  |  |  |  |
| ≤ 0.204 | 19 (31.1%) | 60 (23.8%) |  | Reference |  |  |
| 0.204-0.278 | 22 (36.1%) | 57 (22.6%) |  | 1.190 (0.553-2.560)^b^ | 0.656 |  |
| 0.278-0.361 | 10 (16.4%) | 68 (27.0%) |  | 0.808 (0.424-1.542)^b^ | 0.057 |  |
| > 0.361 | 10 (16.4%) | 67 (26.6%) |  | 0.808 (0.424-1.542)^b^ | 0.056 |  |
| *p* value for trend |  |  |  |  | 0.011^*^ |  |
| **Model 3** | | | | |  |  |
| CCDC80 Z-score |  |  |  | 0.595 (0.387-0.915)^a^ | 0.018^*^ |  |
| CCDC80 (ng/mL) |  |  |  |  |  |  |
| ≤ 0.204 | 19 (31.1%) | 60 (23.8%) |  | Reference |  |  |
| 0.204-0.278 | 22 (36.1%) | 57 (22.6%) |  | 1.319 (0.638-2.723)^b^ | 0.455 |  |
| 0.278-0.361 | 10 (16.4%) | 68 (27.0%) |  | 0.543 (0.228-1.294)^b^ | 0.168 |  |
| > 0.361 | 10 (16.4%) | 67 (26.6%) |  | 0.543 (0.230-1.284)^b^ | 0.165 |  |
| *p* value for trend |  |  |  |  | 0.051 |  |
| **Model 4** | | | | |  |  |
| CCDC80 Z-score |  |  |  | 0.546 (0.356-0.837)^a^ | 0.005^*^ |  |
| CCDC80 (ng/mL) |  |  |  |  |  |  |
| ≤ 0.204 | 19 (31.1%) | 60 (23.8%) |  | Reference |  |  |
| 0.204-0.278 | 22 (36.1%) | 57 (22.6%) |  | 1.257 (0.605-2.611)^b^ | 0.539 |  |
| 0.278-0.361 | 10 (16.4%) | 68 (27.0%) |  | 0.470 (0.202-1.095)^b^ | 0.080 |  |
| > 0.361 | 10 (16.4%) | 67 (26.6%) |  | 0.471 (0.199-1.114)^b^ | 0.086 |  |
| *p* value for trend |  |  |  |  | 0.019^*^ |  |
| **Model 5** | | | | |  |  |
| CCDC80 Z-score |  |  |  | 0.518 (0.326-0.821)^a^ | 0.005^*^ |  |
| CCDC80 (ng/mL) |  |  |  |  |  |  |
| ≤ 0.204 | 19 (31.1%) | 60 (23.8%) |  | Reference |  |  |
| 0.204-0.278 | 22 (36.1%) | 57 (22.6%) |  | 1.034 (0.493-2.169)^b^ | 0.029^*^ |  |
| 0.278-0.361 | 10 (16.4%) | 68 (27.0%) |  | 0.365 (0.148-0.900)^b^ | 0.519 |  |
| > 0.361 | 10 (16.4%) | 67 (26.6%) |  | 0.440 (0.182-1.062)^b^ | 0.068 |  |
| *p* value for trend |  |  |  |  | 0.014^*^ |  |
|  |  |  |  |  |  |  |
|  |  |  |  |  |  |  |

Model 1: Unadjusted; Model 2: Adjusted for age, gestational age, BMI, SBP; Model 3: Adjusted for ALT, AST and creatinine; Model 4: Adjusted for IL-6 and CRP; Model 5: Adjusted for triglyceride, cholesterol, HDL-C, LDL-C, apoA1 and apoB.

^a^ The OR (95% CI) of CCDC80 z-scores using a logistic regression.

^b^ The OR (95% CI) referenced to the lowest level using a logistic regression.

**P* < 0.05

Table S2 Univariate and multivariate regression analyses for unmatched whole subjects

| Variables | Univariate | |  | Multivariate | |
| --- | --- | --- | --- | --- | --- |
|  | r | *P* value |  | *β* (95%*CI*)^a^ | *P* value |
| FBG (mmol/L) | -0.061 | 0.285 |  | -0.146 (-0.449, 0.157) | 0.344 |
| OGTT 1h (mmol/L) | -0.058 | 0.310 |  | -0.460 (-1.567, 0.647) | 0.414 |
| OGTT 2h (mmol/L) | -0.035 | 0.536 |  | -0.242 (-1.110, 0.625) | 0.583 |
| BMI (kg/m^2^) | -0.036 | 0.528 |  | -0.966 (-3.695, 1.763) | 0.487 |
| SBP (mmHg) | -0.011 | 0.847 |  | -0.916 (-9.788, 7.957) | 0.839 |
| DBP (mmHg) | -0.038 | 0.498 |  | -2.072 (-8.127, 3.984) | 0.501 |
| ALT (U/L) | 0.164 | 0.004^*^ |  | 7.825 (2.741, 12.908) | 0.003^*^ |
| AST (U/L) | 0.212 | <0.001^*^ |  | 8.524 (4.226, 12.822) | <0.001^*^ |
| MAO (U/L) | 0.158 | 0.005^*^ |  | 2.302 (0.650, 3.954) | 0.006^*^ |
| Creatinine (umol/L) | 0.081 | 0.152 |  | 15.493 (-13.229, 44.216) | 0.289 |
| Hemoglobin (g/L) | -0.121 | 0.032^*^ |  | -9.650 (-18.773, -0.527) | 0.038^*^ |
| **Inflammatory marker** | | | | | |
| WBC count (× 10^9^/L) | -0.017 | 0.767 |  | -0.763 (-5.067, 3.540) | 0.727 |
| IL-6 (pg/mL) | -0.003 | 0.962 |  | -0.007 (-0.622, 0.609) | 0.983 |
| CRP (mg/L) | -0.096 | 0.091 |  | -1.824 (-3.967, 0.319) | 0.095 |
| Complement C1q (mg/L) | 0.099 | 0.079 |  | 23.278 (-1.891, 48.448) | 0.070 |
| **Lipids** | | | | | |
| Triglyceride (mmol/L) | 0.028 | 0.627 |  | 0.220 (-0.455, 0.895) | 0.521 |
| Cholesterol (mmol/L) | 0.065 | 0.252 |  | 0.796 (-0.571, 2.162) | 0.253 |
| HDL-C (mmol/L) | 0.108 | 0.055 |  | 0.293 (0.001, 0.585) | 0.049^*^ |
| LDL-C (mmol/L) | 0.140 | 0.013^*^ |  | 0.672 (0.143, 1.200) | 0.013^*^ |
| Apolipoprotein A1 (g/L) | 0.171 | 0.002^*^ |  | 0.453 (0.167, 0.739) | 0.002^*^ |
| Apolipoprotein B (g/L) | 0.159 | 0.005^*^ |  | 0.246 (0.080, 0.412) | 0.004^*^ |

BMI body mass index, FBG fasting blood glucose, OGTT oral glucose tolerance test, SBP systolic pressure, DBP diastolic pressure, WBC white blood cell, IL-6 interleukin-6, CRP C-reaction protein, ALT alanine aminotransferase, AST Aspartic aminotransferase, MAO monoamine oxidase, HDL-C high-density lipoprotein cholesterol, LDL-C low-density lipoprotein cholesterol

^a^ adjusted maternal age and Gestational age. **P* < 0.05
