# Supplementary figures and images for: A novel association of CCDC80 with gestational diabetes mellitus in pregnant women: a propensity score analysis from a case-control study
Source: BMC Pregnancy Childbirth. 2020 Jan 28;20:53. doi: 10.1186/s12884-020-2743-3 (PMC6986032; doi:10.1186/s12884-020-2743-3)

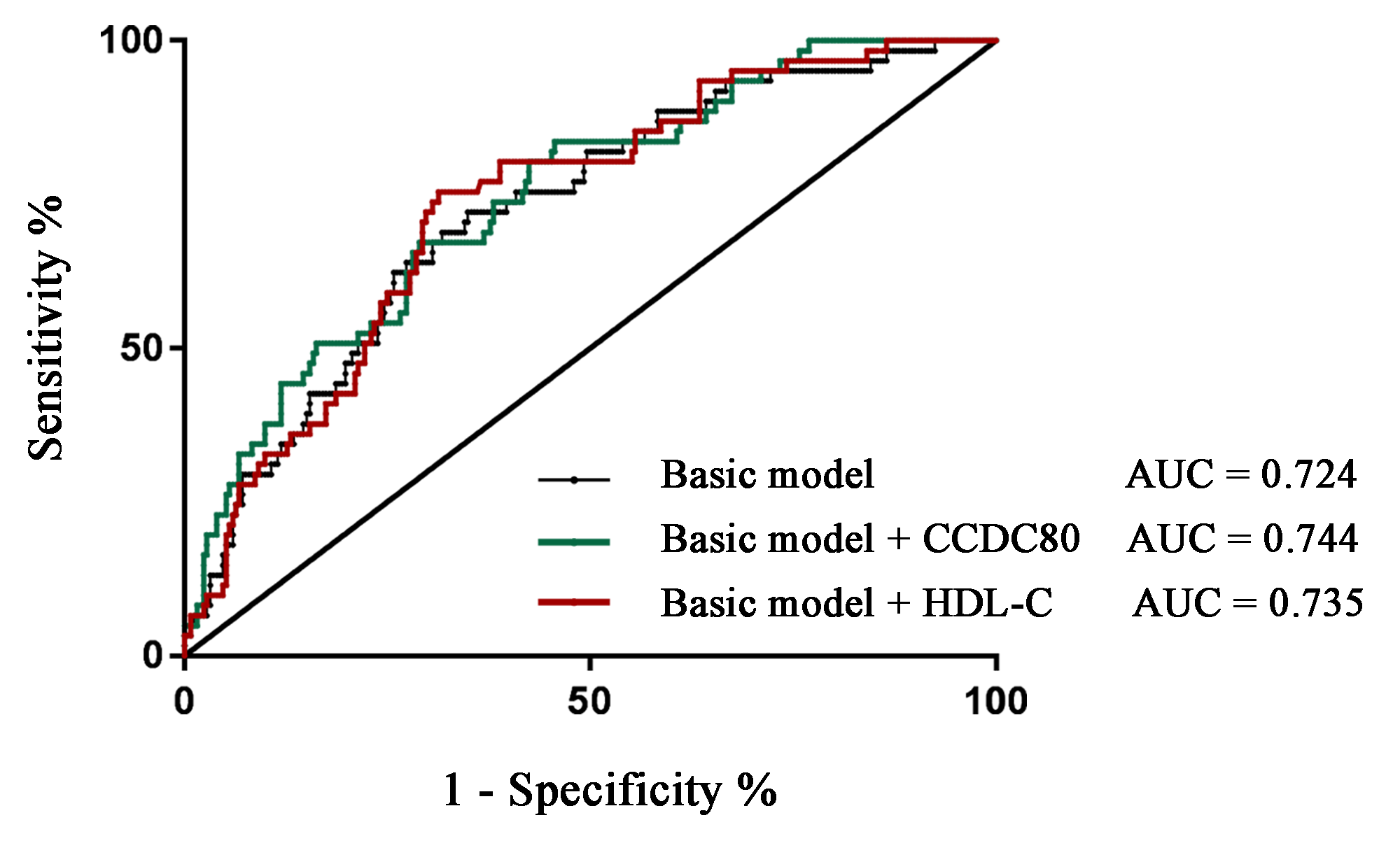

Supplement: Supplementary file 2 — Additional file 2. ROC curves and summaries for all participants using a basic model (including age, gestational age, BMI, SBP, and DBP) and with addition of independent predictors (CCDC80 and HDL-C). [file 12884_2020_2743_MOESM2_ESM.tif]
